# Neuroinflammation and Neuronal Loss Precede Aβ Plaque Deposition in the hAPP-J20 Mouse Model of Alzheimer’s Disease

**DOI:** 10.1371/journal.pone.0059586

**Published:** 2013-04-01

**Authors:** Amanda L. Wright, Raphael Zinn, Barbara Hohensinn, Lyndsey M. Konen, Sarah B. Beynon, Richard P. Tan, Ian A. Clark, Andrea Abdipranoto, Bryce Vissel

**Affiliations:** 1 Neurodegenerative Disorders, Garvan Institute of Medical Research, Neuroscience Department, Sydney, Australia; 2 Faculty of Medicine, University of New South Wales, Sydney, Australia; 3 Research School of Biology, Australian National University, Canberra, Australia; University of New South Wales, Australia

## Abstract

Recent human trials of treatments for Alzheimer’s disease (AD) have been largely unsuccessful, raising the idea that treatment may need to be started earlier in the disease, well before cognitive symptoms appear. An early marker of AD pathology is therefore needed and it is debated as to whether amyloid-βAβ? plaque load may serve this purpose. We investigated this in the hAPP-J20 AD mouse model by studying disease pathology at 6, 12, 24 and 36 weeks. Using robust stereological methods, we found there is no neuron loss in the hippocampal CA3 region at any age. However loss of neurons from the hippocampal CA1 region begins as early as 12 weeks of age. The extent of neuron loss increases with age, correlating with the number of activated microglia. Gliosis was also present, but plateaued during aging. Increased hyperactivity and spatial memory deficits occurred at 16 and 24 weeks. Meanwhile, the appearance of plaques and oligomeric Aβ were essentially the last pathological changes, with significant changes only observed at 36 weeks of age. This is surprising given that the hAPP-J20 AD mouse model is engineered to over-expresses Aβ. Our data raises the possibility that plaque load may not be the best marker for early AD and suggests that activated microglia could be a valuable marker to track disease progression.

## Introduction

Alzheimer’s disease (AD) is a neurodegenerative disorder characterized symptomatically by impaired memory, alterations to personality and decreased visual-spatial skills. Pathologically, AD is characterized by a loss of neurons, central inflammation, amyloid-β (Aβ) aggregation into plaques and by the formation of neurofibrillary tangles (NFTs) consisting of hyperphosphorylated tau [1]. Of these hallmarks, plaque load has historically been regarded as the definitive diagnosis of AD at autopsy of a person who had dementia [2].

Plaques result from the cleavage of the Aβ precursor protein (APP) by β- and γ-secretases into 39–43 amino acid Aβ peptides within the cerebral cortex, hippocampus and amygdala [3], [4]. In the normal state, APP is cleaved to produce a fragment of 40 amino acids in length termed Aβ_40_. However, in AD, cleavage often results in an overproduction of the more fibrillogenic form, Aβ_42_, which can form neuritic plaques [5]. Since plaque load has been regarded as both a hallmark and cause of AD, numerous recent drug trials have focused on reducing fibrillogenic Aβ. Unfortunately, to date these clinical trials have largely failed, raising the notion that the treatments are being delivered too late in the disease progression, and/or that reducing Aβ load may not be the best target for preventing AD progression.

Plaque load has long been considered to be the major hallmark and therapeutic target for AD, and as such it is now being extensively investigated as an early prognostic marker of AD. Consequently, the first FDA-approved Aβ imaging ligand (Amyvid™), which detects neuritic plaques, has recently been released. However, there is still debate as to the clinical relevance of neuritic plaques as the correlation between plaque deposition and cognitive status is not clear [6], [7], [8]. Furthermore, plaques can be detected in people without cognitive deficits indicating that plaque load may not be the most precise biomarker for AD [9], [10].

While the pathognomonic hallmarks of AD include plaques, AD is also associated with NFTs, neuronal loss and increased neuroinflammation [11]. Neuronal loss is usually prominent in the hippocampus, especially the CA1 region, and is further detected throughout the cerebral cortex, increasing with disease progression [12]. In addition, postmortem studies have also demonstrated significant neuroinflammatory changes in brain tissue from AD patients [3]. Microglia, the brain’s local macrophage, and astrocytes are known to produce pro-inflammatory cytokines such as tumor necrosis factor-alpha (TNF-α? and interleukin-6 (IL-6) when activated [13], [14], [15]. These, and other, cytokines have been implicated in neurodegeneration and plaque formation [11], [16]. Despite an understanding that neuroinflammation and neuronal loss contribute to disease progression, the timing of these events is undetermined. It is apparent from the current debate that a full understanding of the time course of AD pathology as it relates to symptoms is required, both to allow accurate diagnosis and to potentially identify events early in disease that could be targeted for treatment.

In this study we therefore aimed to determine the timing of common pathological markers of AD including Aβ oligomer formation, Aβ plaque load, neuronal loss, and neuroinflammation. These classical hallmarks have previously been reported in mouse models of AD that overexpress APP [17], [18], [19], [20], [21]. In particular, the J20 mouse model, generated by Mucke *et al.* (2000) is of interest as it develops early plaque formation from several months of age, has severe synaptic dysfunction, and is susceptible to seizure activity [22], [23], [24], [25], [26], [27]. This line expresses human APP (hAPP) bearing two mutations; the Swedish (K595N) and Indiana (M596L) mutations. However, the timing of pathological events has not been well characterized in the hAPP-J20 mouse model.

In order to address the timing of pathological events, we adopted a highly accurate and unbiased stereological counting method to detect age-dependent changes in the number of neurons, astrocytes, and microglia in the hippocampus of hAPP-J20 mice and their wild-type (WT) littermates. Tau hyperphosphorylation does not occur in these mice and was therefore not investigated in this study [28]. By accurately quantifying cell numbers, we have identified that neuronal cell loss and inflammatory changes occur well in advance of the formation of Aβplaques. Current pharmacological trials are based on reducing plaque load and significant worldwide effort is being made to identify methods of imaging plaques as a marker of disease progression [29], [30]. Our findings therefore have important therapeutic implications because they suggest that plaque load may be among the last events, occurring late in the disease process, after cell loss and inflammatory elevation.

## Methods

### Mice

Male hemizygous transgenic (hAPP-J20) and non-transgenic mice (WT) were from the J20 line, which express h-APP containing both the Swedish and Indiana mutations, under a PDGF-β chain promoter [24]. Mice were housed at a maximum five mice per cage, until the study began, at which time mice were housed individually. Mice were kept on a 12 h light/dark cycle (lights on at 7:00 am). Food and water were available *ad libitum* until dietary restrictions began. All animal experiments were performed with the approval of the Garvan Institute and St. Vincent’s Hospital Animal Ethics Committee, in accordance with National Health and Medical Research Council animal experimentation guidelines and the Australian Code of Practice for the Care and Use of Animals for Scientific Purposes (2004).

### Immunofluorescence

Mice were anesthetized with ketamine (8.7 mg/mL) and xylazine (2 mg/mL) and transcardially perfused with 4% paraformaldehyde (PFA). Brains were harvested and postfixed in 4% PFA for 6 h before being transferred to 30% sucrose. Brains were sectioned coronally (40 µm) with a cryostat. Free-floating sections were used. For the detection of Aβ oligomers, sections were washed three times in phosphate buffered saline (PBS) and incubated for 1 h at room temperature in 15% Fetal Bovine Serum (FBS) +0.1% Triton-X 100 (TX-100) blocking solution. Sections were incubated overnight at 4°C in the anti-Aβ oligomer antibody, A11 (1:100, Millipore). Sections were washed three times in PBS and incubated in Alexa Fluor 594 Goat anti-rabbit IgG (1:250, Invitrogen) for 3 h at room temperature. Sections were mounted and cover slipped with Kaisers glycerol gelatin solution (Merck). Slides were imaged using a Zeiss Axioplan upright fluorescence microscope with Zeiss Axiocam MRm digital camera. Digital images were captured using Axiovision V 4.8.1.0 software.

### Immunohistochemistry

Endogenous peroxidases were quenched using 3% H_2_0_2_ and were subjected to blocking with 3% Bovine Serum Albumin (BSA) +0.25% TX-100 in PBS. Sections were incubated in the following primary antibodies: mouse anti-NeuN (1:500; Chemicon), rat anti-CD68 (1:100; AbD Serotec), and rabbit anti-GFAP (1:300; Dako) for 72 h at 4°C followed by biotin-labeled secondary antibodies, HRP-labeled avidin-biotin complex and 3,3′-Diaminobenzidine (DAB). Sections stained for CD68 and GFAP were counterstained with mouse anti-NeuN that was detected with Nova-Red to outline the region of interest. Sections were mounted and cover slipped with Kaisers glycerol gelatin solution (Merck). For detection of total Aβ, sections were incubated in a biotinylated 6E10 antibody (1:1000, Covance) for 24 h at 4°C and subjected to HRP-labeled avidin-biotin complex and DAB.

### Quantification of Total Aβ

Quantification of the 6E10 staining was performed using the Image-Pro Plus v.6.0 image analysis system to analyze the percent area occupied by positive staining. Images from the hippocampal region subfield at the antero-posterior (AP) positions from bregma between −1.34 mm and −2.3 mm were collected at 10×magnification (five sections per animal). Captured images were imported into Image-Pro Plus and an intensity threshold level was set to allow for the discrimination between 6E10 positive staining and background labeling. The percentage of positive staining was calculated as the Aβ deposition load.

### Aβ Plaque Quantification

Thioflavine S staining was used to determine fibrillar Aβ plaque deposition. Sections were slide mounted and allowed to dry, prior to being washed with distilled water and treated with 70% and 80% EtOH for five minutes. Slides were incubated for 15 minutes with 1% thioflavine S in 80% ethanol. Plaque counts were conducted in the hippocampal region subfield from five sections per animal at the antero-posterior (AP) positions from bregma between −1.34 mm and −2.3 mm. All plaque counts were conducted manually and were blind to genotype and age. Slides were imaged using a Zeiss Axioplan upright fluorescence microscope with Zeiss Axiocam MRm digital camera. Digital images were captured using Axiovision V 4.8.1.0 software.

### Dot Blot

Mice were cervically dislocated and the hippocampus was rapidly dissected from the brain of hAPP-J20 and WT mice and frozen at −80°C until use. Tissue was homogenized in RIPA buffer supplemented with protease inhibitors. Protein concentrations of the supernatant were measured using a Bradford assay and samples were adjusted to the same concentrations with SDS buffer. 20 µg of extract was applied to a nitrocellulose membrane and air-dried. Membranes were incubated in a 10% solution of nonfat dry milk for 1 h at room temperature and overnight at 4°C in A11 (1:1000, Millipore). Membranes were then washed, before being incubated in HRP-conjugated secondary and visualized by ECL. Films were scanned and Aβ oligomer levels were quantified using Image J Software. For quantification of dot blots, the raw values obtained from hAPP-J20 mice were adjusted with the values obtained from the WT mice.

### Aβ ELISA

Hippocampi from hAPP-J20 mice were weighed and homogenized in 5vol/wt of Tris-buffered saline (TBS) (Tris-HCL 50 mM pH 7.6; NaCl 150 mM; EDTA 2 mM) containing a cocktail of protease inhibitors. Samples were then suspended in 2% SDS containing protease inhibitors and centrifuged at 100,000 *g* for 60 minutes at 4°C. The supernatant was collected for the soluble Aβ ELISA. The Aβ levels were determined by using the commercially available BetaMark Total Beta-Amyloid Chemiluminescent ELISA Kit (Covance).

### Stereology

Quantification of cell population estimates were made using Stereo Investigator 7 (Microbrightfield) as previously described [31], [32]. Estimates were conducted on the dorsal hippocampus at the antero-posterior (AP) positions from bregma between −1.34 mm and −2.3 mm. For neuronal population estimates, a minimum 20 sampling sites were sampled per section on a grid size of 84 µm×60 µm and a counting frame size of 30 µm×30 µm. For GFAP-positive astrocyte population estimates, a minimum of 30 sampling sites per section on a grid size of 68 µm×68 µm and a counting frame size of 30 µm×30 µm. For CD68-positive microglial population estimates, a minimum of 40 sampling sites were sampled per section on a grid site of 114 µm×68 µm and a counting frame size of 65 µm×65 µm. For all cell population estimates, a guard zone of 5 µm and a dissector height of 10 µm were used. Each marker was assessed at one in every sixth section, with a total of five sections being sampled. The regions sampled included the CA3 and CA1 regions of the hippocampus for neuronal and astrocyte populations. Microglia populations were sampled within the borders of the CA1, CA3 and dentate gyrus (DG) regions of the hippocampus. All stereological cell counts were performed blind to genotype and age.

### Behavior

#### Open field test

The open field test arena (40×40 cm) was situated in a large box with clear plexiglass walls, no ceiling, and a white floor. Each chamber was set inside a larger sound-attenuating cubicle with lights illuminating the arena and a fan to eliminate background noise. Mice were placed into the center of the arena and allowed to explore the test box for 10 minutes, while a computer software program (Activity Monitor; Med Associates) recorded activity via photobeam detection inside the testing chambers as a measure of general activity levels. The total distance traveled over the course of the 10 minutes was recorded. The arena was cleaned with 70% ethanol (EtOH) between each mouse.

#### Elevated plus test

The elevated plus-maze consists of four arms (77×10 cm) elevated (70 cm) above the floor. Two of the arms contained 15 cm-high walls (enclosed arms) and the other two consisted of no walls (open arms). Each mouse was placed in the middle of the maze facing a closed arm and allowed to explore the maze for five minutes. A video camera recorded the mouse and a computer software program (Limelight; Med Associates) was used to measure the time spent in the open arms, as an indication of anxiety-like behavior. The maze was cleaned with 70% EtOH between each mouse.

#### Radial arm maze

The radial arm maze (RAM) consists of eight arms (65×9 cm), extending radially from a central arena (35 cm diameter), elevated (90 cm) above the ground. Each arm and the central arena were made of plexiglass, with enclosing walls made of clear plexiglass. The RAM was cleaned with 70% EtOH between each mouse.

Mice were individually housed and restricted to 85% of their original body weight for one week prior to the commencement of RAM testing. On the first and second day, mice were habituated to the maze by being placed into the central arena, with each of the eight arms baited with sweetened condensed milk, and were allowed to explore the maze for 10 minutes. Starting on the third day, and continuing for 24 days twice a day, mice were subjected to a reference memory task, where the same three of the eight arms were baited with sweetened condensed milk. The training trial continued until all three baits were retrieved or until five minutes had elapsed. After a 14-day rest period mice were presented to a retention trial where the same arms were baited with sweetened condensed milk.

An investigator recorded measures, with the number of successful entries into the baited arms (where the sweetened condensed milk was consumed) being divided by the total number of entries made. Data is presented as “Session”, consisting of two days (a total of four trials).

### Fear Conditioning

Training and testing took place in two identical cube-shaped fear-conditioning chambers (32×27×26 cm; Med Associates Inc.) that had a clear plexiglass door, ceiling and rear wall and grey aluminum side walls. Each chamber had a removable grid floor, which consisted of 36 parallel rods spaced 8 mm apart. Positioned under the grid was a removable grey aluminum tray for collection of waste. The rods were connected to a shock generating and scrambling system, which delivered a current to elicit a foot shock. This system was connected to and controlled by computer software (FreezeFrame2, Actimetrics). A video camera, which was positioned in front of the chambers, recorded the behavior of the mice during training and testing. The fear-conditioning chamber was cleaned with 70% EtOH and the waste tray was scented with aniseed essence between each mouse.

On the conditioning day, mice were placed into a fear-conditioning chamber in which the environment (context) was controlled. Mice were allowed to explore the context freely for 1 minute prior to receiving a single moderate footshock (0.5 mA, 2s). Following shock, all mice remained in the chamber for 30 seconds and were then immediately returned to their homecages. On the following day, the mice were exposed to the same context and behavior was recorded for three minutes.

Freezing was assessed as a measure of fear on all days using a 4 second sampling method by investigators, who were blind to the genotype. The number of observed freezes was averaged and divided by the total number of samples taken to yield a percentage of freezing. Data is presented as the average percentage of freezing during the three minutes test period.

### Statistical Analysis

All statistical analysis was performed using the statistical package SPSS v19 (Graduate pack) (SPSS Inc., Chicago, IL, http://www.spss.com). Differences between means were assessed, as appropriate, by one- or two- way ANOVA with or without repeated measures, followed by Bonferroni *post hoc* analysis. Correlations were assessed by simple linear regression. For behavioral studies, experiments were conducted three times for correct statistical approach [33].

## Results

### Aβ Expression and Plaque Formation Occurs in an Age-Dependent Manner

**Figure 1:**
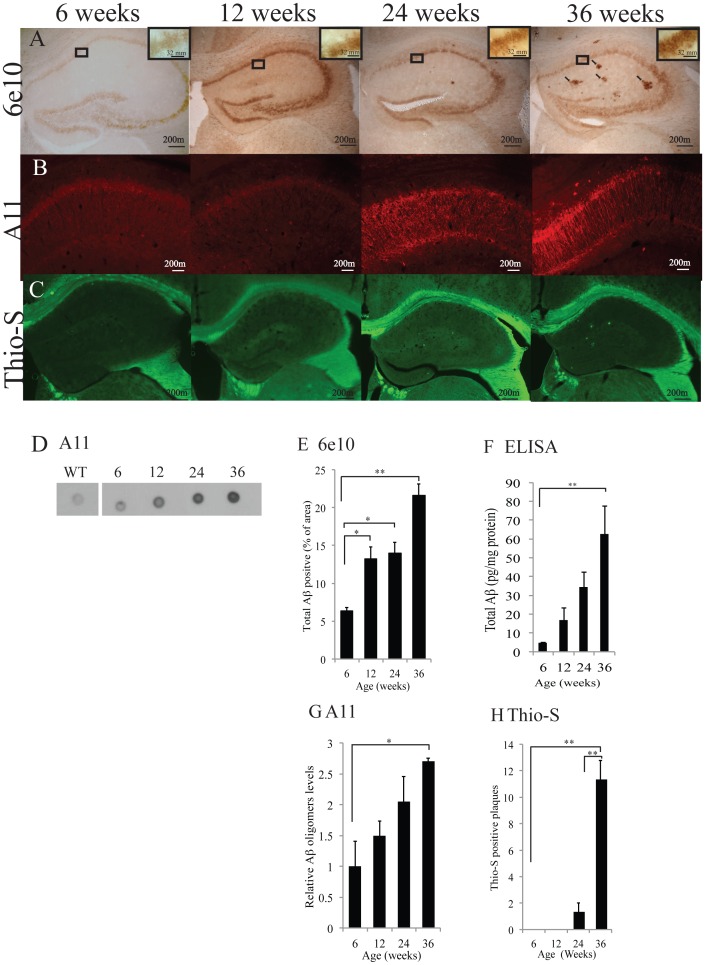
Age-dependent Aβ expression and plaque deposition in the hAPP-J20 mice. (A) 6E10 immunohistochemistry illustrated increased neuronal Aβ from 6 to 12, 24 and 36-week-old hAPP-J20 mice (quantified in E). (B) Aβ oligomer formation was not apparent until 24 weeks of age and appeared by 36 weeks of age when it appeared to be associated with neuronal processes. (C) Plaques were present by 36 weeks of age, but not earlier (quantified in F). (D and G) A dot plot quantification with the Aβ-oligomer specific antibody, A11, revealed increases in Aβ oligomers through aging in the hAPP-J20 mouse, with a significant increase in 36-week-old hAPP-J20 mice. (H) Quantification of Aβ by ELISA revealed an increase in total Aβ from 6 (p<0.05) and 12 (p<0.05) to 36 weeks of age in the hAPP-J20 mouse. Each value represents the mean ± standard error of the mean (SEM). *p<0.05, **p<0.01, ***p<0.001.

An important characteristic of AD is the accumulation of the protein Aβ and the resulting formation of plaques throughout the brain. To determine whether hAPP-J20 mice exhibit age-dependent accumulation of cellular and extracellular Aβ, we measured total Aβ using immunohistochemical techniques with the 6E10 antibody in mice of different ages. We observed neuronal Aβ throughout the hippocampus at 6, 12, 24 and 36 weeks (Figures 1A) Quantification of 6E10 immunoreactivity revealed a significant increase in total Aβlevels with age (Figure 1E; *F*_(3,24)_ = 23.14 *p*<0.001). A Bonferroni *post-hoc* analysis revealed a significant increase in Aβ at 12 (*p*<0.05), 24 (*p*<0.05) and 36 weeks (*p*<0.001) when compared to 6 weeks (Figure 1E). Hippocampal oligomeric Aβ expression also increased in an age-dependent manner, and was significantly present by 36 weeks of age (p<0.05; Figures 1C, 1D and 1G) and interestingly appeared to form along the axons of neurons (Figure 1C). In addition to total and oligomeric Aβ, a significant number of plaques were present at 36 weeks of age (*p*<0.001) (Figure 1B and 1F). This indicates that the rise in hippocampal monomeric and oligomeric Aβ precedes plaque formation by a significant margin, as described in other models of AD [34], [35].

We further determined buffer-soluble hippocampal Aβ in hAPP-J20 mice at 6, 12, 24 and 36 weeks of age by a total Aβ sandwich ELISA. This showed a significant increase in total Aβ levels with age (Figure 1H; *F*_(3,24)_ = 7.761 *p*<0.001). A Bonferroni *post-hoc* analysis revealed a significant difference between 6 (*p*<0.001) and 12 weeks (*p*<0.05), when compared to 36 weeks of age. Combined, these results demonstrate age-dependent expression of Aβ that is followed by senile plaque formation at later stages in the hippocampus of hAPP-J20 mice.

### hAPP-J20 Mice Exhibit Loss of Neurons in the CA1, but not CA3, Region of the Hippocampus

**Figure 2:**
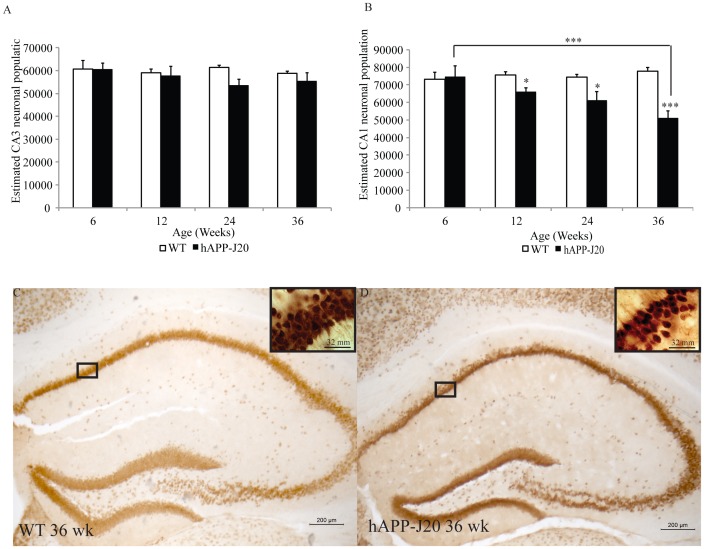
Quantification of hippocampal neuronal populations in hAPP-J20 mice. (A) No cell loss was detected in the CA3 region of the hippocampus of hAPP-J20 mice at 6, 24 and 36 weeks of age. (B) No cell loss in the CA1 region was detected at 6 weeks, however, 12 (*p*<0.05), 24 (*p*<0.05) and 36-week-old (*p*<0.001) mice showed significant cell loss when compared to aged-matched WT controls. Moreover, cell loss was significantly different between 6 and 36-week-old hAPP-J20 mice (*p*<0.001). Cell loss in the CA1 region can be qualitatively seen between (C) WT and (D) 36-week-old hAPP-J20 mice. Each value represents the mean ± standard error of the mean (SEM). *p<0.05, ***p<0.001.

Given the high abundance of Aβ in the hippocampus of hAPP-J20 mice starting at 6 weeks of age, and its association with cell loss in other models [20], [36], we hypothesized that Aβ expression would be associated with neurodegeneration in the hAPP-J20 mouse model. To examine whether hAPP-J20 also exhibits age-dependent neuronal cell loss in the CA3 and CA1 regions of the hippocampus, we performed unbiased stereological cell counts of NeuN-labelled neurons under brightfield microscopy. Interestingly, analysis of the neuronal population in the CA3 regions of the hippocampus (Figure 2A) demonstrated no significant age-dependent neuronal cell loss from 6, 12, 24 and 36 weeks of age (interaction (*F*_(7,29)_ = 0.783 *p* = 0.514); age (*F*_(7,29)_ = 0.645 *p* = 0.593); genotype (*F*_(7,29)_ = 2.107 *p* = 0.158)).

However, we observed a significant genotype by age interaction in the CA1 region of the hippocampus (*F*_(7,29)_ = 5.264 *p*<0.01; Figure 2B), thus suggesting age-dependent progressive loss of neurons. Therefore, separate one-way ANOVAs were conducted on genotype and age. Six-week-old hAPP-J20 mice did not show neuronal cell deficits in the CA1 as compared to their age-matched WT littermates. However, in contrast, significant neuronal loss in the CA1 was observed in 12 (*F*_(1,8)_ = 6.930 *p*<0.05), 24 (*F*_(1,8)_ = 6.966 *p*<0.05) and 36 week (*F*_(1,8)_ = 33.537 *p*<0.001; Figure 2C and 2D) old hAPP-J20 mice, when compared to their age-matched WT controls. A one-way ANOVA of genotype indicated significant cell loss in the CA1 of hAPP-J20 mice with age (*F*_(3,14)_ = 4.807 *p* = 0.017). A Bonferroni *post-hoc* analysis revealed a significant difference in neuronal cell population between 6 week and 36-week-old hAPP-J20 mice (*p*<0.001). Additionally, there was a significant correlation of neuronal cell loss and total Aβ expression (Table 1; *p*<0.01). This is consistent with the idea that Aβ may be playing a direct or indirect role in cell death in the CA1 region of the hippocampus, or, in theory, that cell death is playing a role in Aβ accumulation.

**Table 1:** Correlations between CA1 regions (NeuN and GFAP), CD68 cell numbers and total hippocampal Aβ.

	NeuN	GFAP	CD68
Aβ	*r* = 0.77**	*r = *0.57*	*r = *0.66*
CD68	*r = *0.80**	*r = *0.42	
GFAP	*r* = 0.38		

* *p*<0.05.

** *p*<0.01.

### hAPP-J20 Mice Exhibit an Increased Astrocyte Population, Reaching Saturation at 24 Weeks

**Figure 3:**
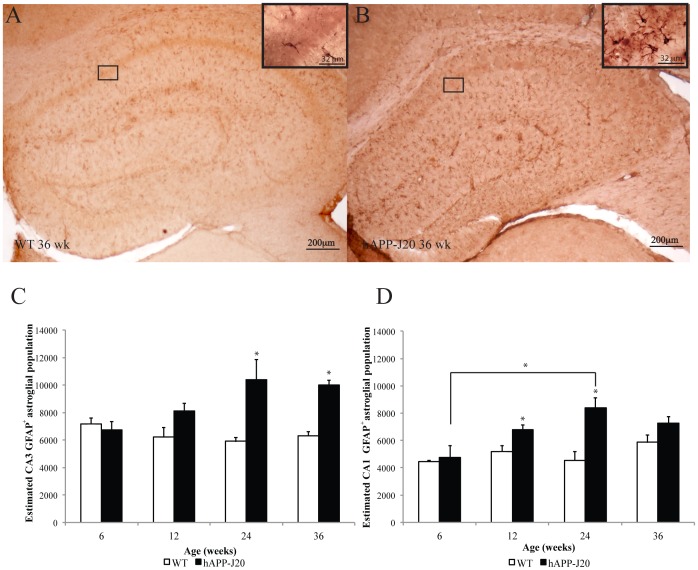
Quantification of GFAP-positive astrocytes in hAPP-J20 mice. GFAP-positive astrocytes in the hippocampus were observed more often in (B) 36-week-old hAPP-J20 mice compared to age-matched (A) WT littermates. (C) Quantification analysis revealed no differences in GFAP-positive astrocytes in the CA3 at 6 or 12 weeks, however significant increases in cell number were detected at 24 (*p*<0.05) and 36 weeks (*p*<0.05). (D) In the CA1 region of the hippocampus, there was no increase in GFAP-positive astrocytes at 6 and 36 weeks, though significant increases at 12 (*p*<0.05) and 24 weeks (*p*<0.05) were observed when compared to WT controls. In addition, a significant increase occurred between 6 week and 24-week-old hAPP-J20 mice (*p*<0.05). Each value represents the mean ± standard error of the mean (SEM). *p<0.05.

Gliosis is a hallmark of AD and is characterized by the presence of activated astrocytes. Astrocyte activation results in morphological changes, including the shortening and thickening of processes, increased proliferation and the release of pro-inflammatory factors [11]. To determine the number of glial cells in the hippocampus of the hAPP-J20 mouse model, we performed stereological cell counts in the CA3 and CA1 regions of the hippocampus for astrocyte cells that express the typical marker, GFAP. Our results show that at 36 weeks of age (Figure 3A), hAPP-J20 possessed more gliotic astrocytes when compared to age-matched WT mice (Figure 3B). There was a significant interaction effect of genotype by age for the CA3 region (*F*_(7,29)_ = 4.013 *p* = 0.021; Figure 3C). Therefore, the effect of genotype and age on glial populations in the CA3 was analyzed separately using a one-way ANOVA. Significant differences were apparent in hAPP-J20 mice that were 24 weeks (*F*_(1,8)_ = 9.454 *p*<0.05) and 36 weeks old (*F*_(1,8)_ = 61.728 *p*<0.001) as compared to age-matched WT controls. There was a trend towards significance of age in the CA3 (*F*_(1,14)_ = 3.197 *p* = 0.06) indicating that increased gliotic astrocytes may be age-dependent in hAPP-J20 mice.

Results were similar in the CA1 region of the hippocampus, where there was a significant genotype by age interaction effect (*F*_(7,29)_ = 4.013 *p* = 0.021; Figure 3D). A one-way ANOVA of genotypes revealed significant differences in the number of gliotic astrocytes at 12 (*F*_(1,8)_ = 7.862 *p*<0.05) and 24 weeks of age (*F*_(1,8)_ = 15.478 *p*<0.01), though interestingly not at 36 weeks of age. There was an overall significant age effect on the number of gliotic astrocytes in the CA1 region of the hippocampus of hAPP-J20 mice (*F*_(3,14)_ = 5.722 *p*<0.05). A Bonferroni *post-hoc* analysis revealed a significant difference between 6 weeks and 24 weeks of age (*p*<0.05). In addition, astrocyte numbers in the CA1 region correlated significantly with total Aβ levels (Table 1; *p*<0.05). Combined, these results indicate that increases in reactive astrocyte numbers in the hAPP-J20 mouse model is progressive with age, though peaks at 24 weeks of age.

### Microglial Activation Precedes Amyloid Plaque Deposition

**Figure 4:**
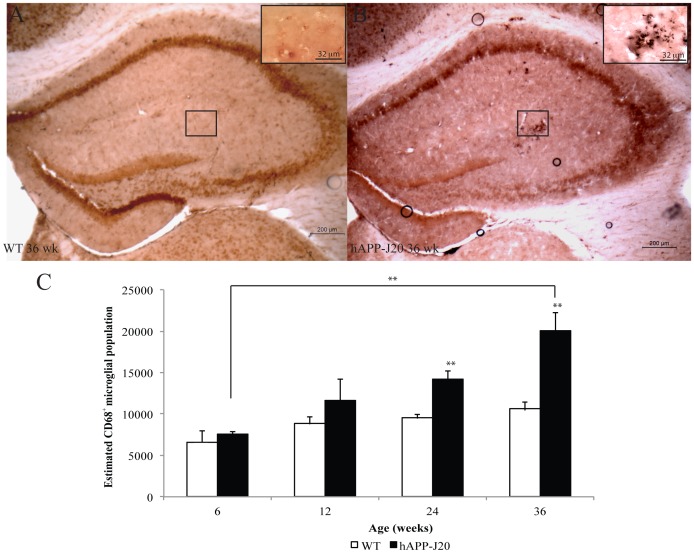
Quantification of CD68-positive activated microglia in hAPP-J20 mice. CD68-positive microglia were observed in the hippocampus of (A) WT mice compared to (B) their hAPP-J20 littermates at 36 weeks of age. Quantification of CD68-positive cell numbers revealed significant increases in cell numbers at 24 (*p*<0.01) and 36 weeks of age (*p*<0.01) in hAPP-J20 mice compared to their age-matched WT littermates. Further, a significant increase in CD68 microglia occurred between 6 week and 36-week-old hAPP-J20 mice (*p*<0.01). Each value represents the mean ± standard error of the mean (SEM). **p<0.01.

Microglial activation has been studied extensively in both mouse models and patients of AD [16]. Microglial activation is characterized by morphological changes from ramified (quiescent) morphology to amoeboid (activated) morphology, the release of pro-inflammatory cytokines and increased microglial cell number. In addition, activated microglia express the marker CD68. As an indicator of increased inflammation we analyzed brain tissue from hAPP-J20 mice for changes in the number of CD68-positive activated microglial cells in the area of the hippocampus bordered by the CA1, CA3 and DG regions of the hippocampus. CD68-positive microglia can be observed in clusters in the hAPP-J20 (Figure 4B) when compared to WT (Figure 4A) mice at 36 weeks of age.

Unbiased stereology was adopted to count CD68 positive microglia. A two-way ANOVA of genotype and age revealed an interaction effect (*F*_(7,29)_ = 5.264 *p*<0.05) on the number of activated microglia in the hippocampus. Therefore, one-way ANOVAs were performed separately on genotype and age. A one-way ANOVA of genotypes revealed significant differences at 24 (*F*_(1,8)_ = 25.298 *p*<0.01) and 36 weeks of age (*F*_(1,8)_ = 23.425 *p*<0.01), but not at 6 and 12 weeks of age when compared to age-matched WT littermates. There was an overall significant age effect (*F*_(3,11)_ = 6.470 *p*<0.01). A Bonferroni *post-hoc* analysis revealed a significant difference between 6 weeks and 36 weeks of age (*p*<0.01). This indicates that activated microglia increase with age in the hAPP-J20 mouse models of AD.

As found with reactive astrocytes, microglia cell populations correlated with the expression of total Aβ (Table 1; *p*<0.05). In addition, microglia cell populations inversely correlated with the number of neurons in the CA1 region of the hippocampus (p<0.05). This shows that activated microglia populations are closely associated with Aβ expression and neuronal cell loss.

### hAPP-J20 Mice Exhibit Hyperactivity, but no Differences in Anxiety

**Figure 5:**
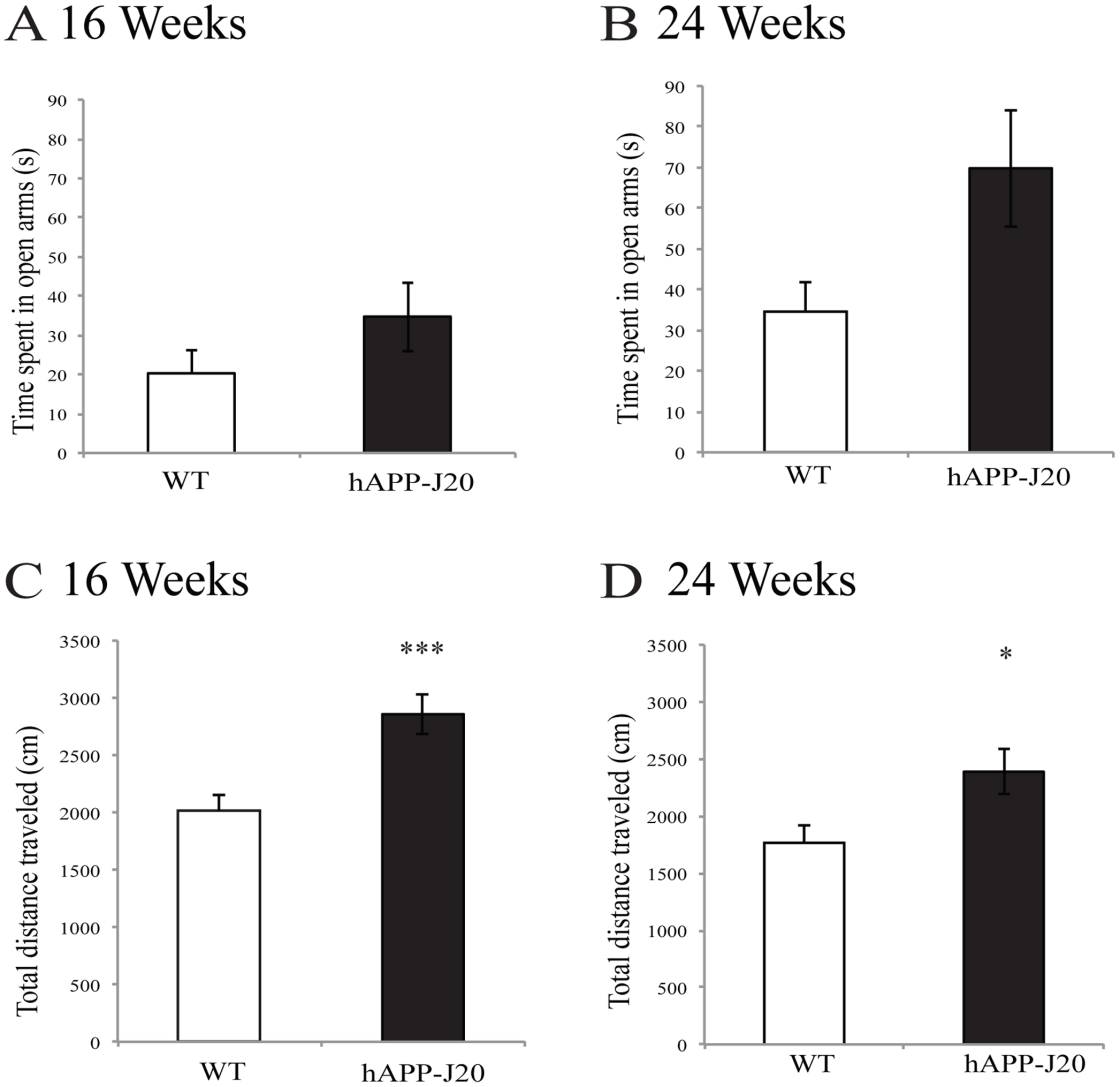
hAPP-J20 mice exhibit hyperactivity. hAPP-J20 mice did not spend significantly more time in the open arm of the elevated plus maze at (A) 16 or (B) 24 weeks of age indicating no difference in anxiety levels compared to age-matched WT littermates. However, hAPP-J20 mice did show hyperactivity at (C) 16 and (D) 24 weeks of age as indicated by the total distance traveled in the open field test. Each value represents the mean ± standard error of the mean (SEM). *p<0.05, ***p<0.001.

In addition to anatomical changes, AD patients and mouse models of AD exhibit profound behavioral alterations [37], [38]. We tested behavioral changes in the hAPP-J20 mouse model using the elevated plus maze and open field test. The elevated plus maze and open field tests are often used as a measure of anxiety and motor activity, respectively. Several studies have indicated that hAPP-J20 mice have increased motor activity and spend more time in the open arm of the elevated plus maze than WT controls, indicating hyperactivity and lower levels of anxiety [28], [39], [40], [41], [42]. In contrast, we found that although the hAPP-J20 mice tend to spend more time in the open arms than the WT controls at 16 (*F*_(1,21)_ = 1.97, *p* = 0.176) and 24 weeks of age (*F*_(1,12)_ = 6.024, *p* = 0.073), it is not significant (Figures 5A and 5B). However, as shown in Figures 5C and 5D, locomotor activity in hAPP-J20 was significantly increased at 16 weeks (*F*_(1,21)_ = 13.91, *p*<0.001) and 24 weeks of age (*F*_(1,12)_ = 6.024, *p*<0.05) compared to WT controls. Combined, these results indicate that hAPP-J20 mice exhibit significantly increased levels of locomotor activity and no changes in anxiety during later stages of AD progression.

### hAPP-J20 Mice Show Spatial Reference Memory Deficits at 16 and 24 Weeks of Age

**Figure 6:**
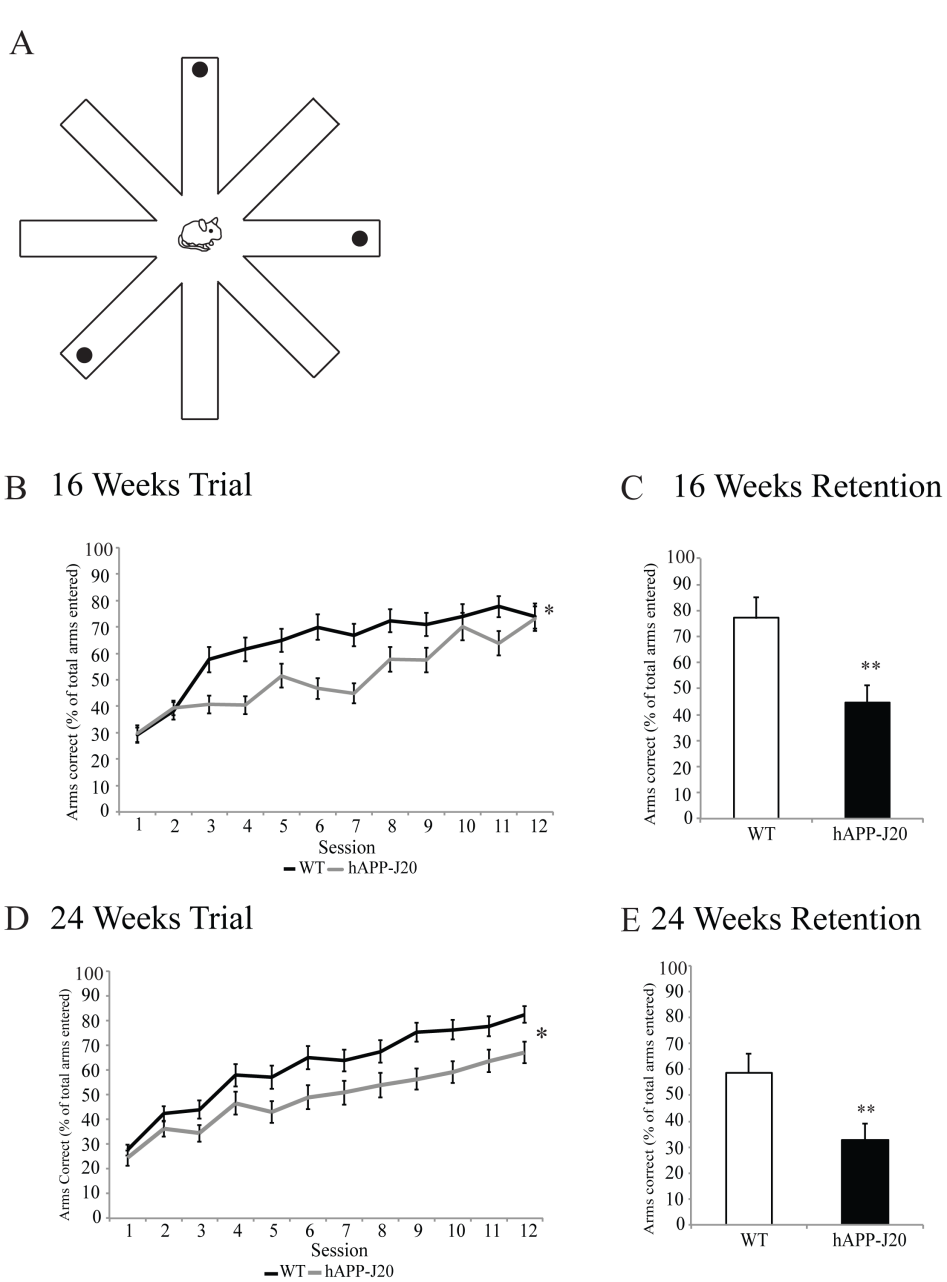
Spatial learning and memory deficits in hAPP-J20 mice. (A) Schematic representation of the radial arm maze. Filled circles represent the baited arms (B) hAPP-J20 mice had significantly impaired spatial reference memory and learning at 16 weeks of age (*p*<0.05) when compared to age-matched WT littermates. (C) 16-week-old hAPP-J20 mice had significant deficits in spatial reference memory and learning retention (*p*<0.05) when compared to age-matched WT littermates. (D) 24-week-old hAPP-J20 mice also showed significantly impaired spatial reference memory and learning (*p*<0.05) when compared to age-matched WT littermates. (E) Spatial reference memory and learning retention was significantly impaired in 24-week-old hAPP-J20 mice (*p*<0.05). Each value represents the mean ± standard error of the mean (SEM). *p<0.05, **p<0.01.

AD is an amnesic disorder and is often associated with profound memory loss [38]. It has been shown that in hAPP-J20 mice, deficits in spatial memory and learning appear as the mice age [39], [43], [44], [45]. A powerful tool for measuring spatial memory and learning is the RAM. Within the RAM, mice use spatial cues to find the hidden food reward (Figure 6A). By using a reference memory version of the RAM, we determined whether hAPP-J20 mice exhibit spatial memory and learning deficits at 16 (Figure 6B–C) and 24 weeks of age (Figure 6D–E). An ANOVA with repeated measures of 16-week-old hAPP-J20 mice and WT mice revealed a significant genotype effect, trial, and a genotype by trial interaction in reference memory (*p*<0.05; Figure 6B). These results indicate that 16-week-old hAPP-J20 mice demonstrate spatial reference memory deficits. As expected, we observed similar deficits in spatial reference memory in 24-week-old hAPP-J20 mice (*p*<0.05; Figure 6D).

Following a 14-day rest period, a retention test was performed. Deficits in retention were detected in both 16 (Figure 6C; *F*_(1,11)_ = 8.22, *p*<0.05) and 24 weeks of age (Figure 6E; *F*_(1,16)_ = 4.65, *p*<0.05) hAPP-J20 mice as compared to age-matched WT controls. These results demonstrate that hAPP-J20 mice exhibit long-term spatial memory and learning deficits.

### hAPP-J20 Mice do not Show a Deficit in Contextual Fear Conditioning

**Figure 7:**
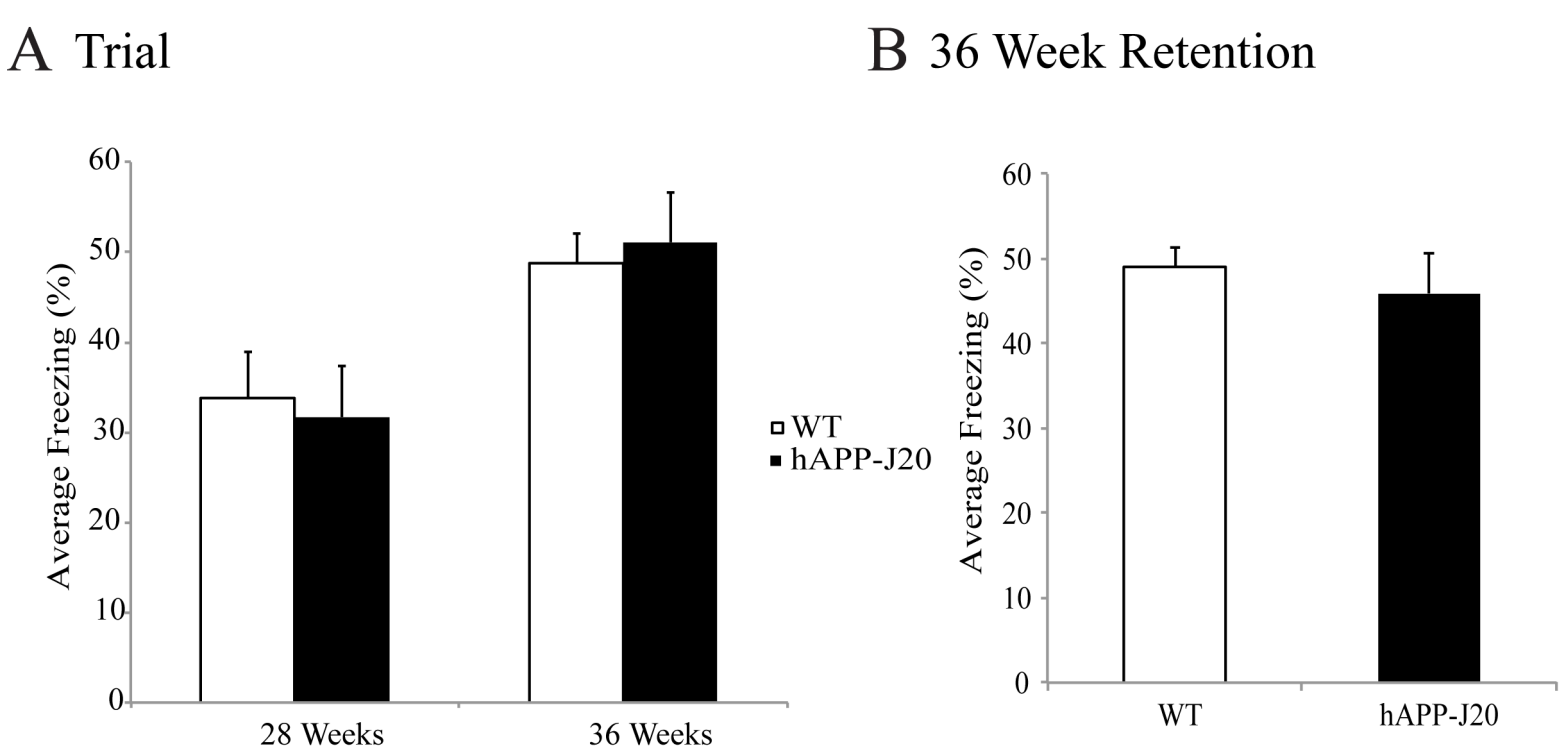
Contextual fear conditioning is not impaired in hAPP-J20 mice. (A) No deficits were seen in the percentage of freezing in 28 and 36-week-old hAPP-J20 mice when compared to age-matched WT littermates. (B) A retention test, performed at 40 weeks, also revealed no deficits in the percentage of freezing in 36-week-old hAPP-J20 mice when compared to age-matched controls. Each value represents the mean ± standard error of the mean (SEM).

There is a vast amount of evidence to show contextual memories are hippocampal-dependent [46]. As such, contextual fear conditioning offers a valuable tool to assess both short-term and long-term memory. Fear conditioning deficits have been detected in other mouse models of AD [47], [48], though not in the hAPP-J20 mouse line [43]. Since we detected neurodegeneration and deficits in spatial learning, we hypothesized contextual fear memory and learning may also be impaired in the hAPP-J20 mouse model of AD. Figure 7A shows there is no difference in freezing behavior at 28 weeks of age (F_(1,15) = _1.308, *p* = 0.7321) as compared to age-matched WT mice. In addition, mice assessed at the 36 weeks of age also did not show a difference in freezing behavior (F_(1,27)_ = 0.433, *p* = 0.511) as compared to age-matched WT mice. In order to determine if long-term memory was impaired in these mice, a retention test was performed on 36-week-old mice, 28 days after their original training in the paradigm. Somewhat surprisingly,at 40 weeks of age, no differences occurred in the long-term retention test (F_(1,27) = _0.140, *p* = 0.711; Figure 7B).

## Discussion

It has recently been suggested that successful treatment of AD may require early intervention. This requires early diagnosis, which in turn depends on identifying early pathological hallmarks of disease. We therefore aimed to identify cellular correlates of early AD in an APP overexpressing mouse, known as the hAPP-J20 mouse model. These mice show plaque formation by seven months of age but, interestingly, no tau hyperphosphorylation at any of the major phosphorylation sites [24], [28]. Our data indicates that AD pathology including neuronal loss, inflammation and behavioral impairment all occurs well before the formation of Aβ plaques, indicating that plaque load may not be the best early diagnostic marker of AD. Therefore, other markers of disease may need to be explored to track the progression of AD.

**Figure 8:**
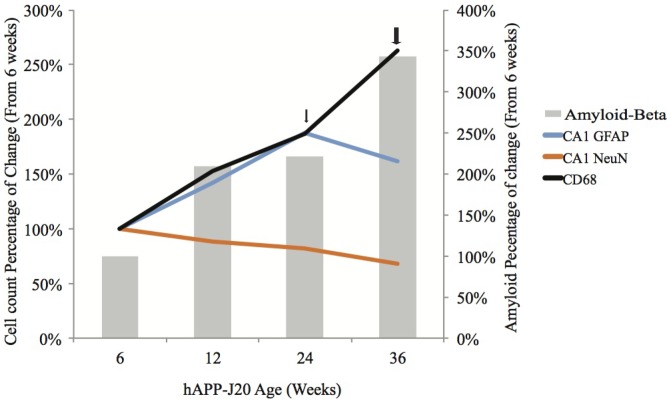
Time course of disease progression, as a percentage of hAPP-J20 6-week-old mice. Mice exhibit 32% loss of neurons in the CA1 region of the hippocampus between 6 weeks and 36 weeks of age. In addition, a 163% increase in the number of CD68-positive microglia and a 62% increase in the number of CA1 GFAP-positive astrocytes occurred between 6 weeks and 36 weeks of age. Total Aβ expression increases by 242% between the ages of 6 weeks and 36 weeks of age. Small arrow represents plaque load in some mice, while larger arrow represents plaque load in all mice.

Neurodegeneration has been described in many mouse models of AD [19], [20], [49], [50] as well as AD patients [51]. However, previous studies have suggested that neuronal loss does not occur in the hAPP-J20 mouse line [52]. Our unbiased accurate estimate of neuronal numbers in these mice revealed a progressive, age-dependent neurodegeneration in the CA1 region, beginning at 12 weeks and reaching a 32% loss by 36 weeks (Figure 8). Interestingly, cell loss does not occur in the CA3 region. This selective loss of CA1 neurons parallels studies of human AD patients that show greater neuron loss in the CA1 compared to the CA3 region [53], [54]. Though the exact reasons for this regional difference are unknown, they may be due to differential expression of both NMDA and AMPA receptor subunits, rendering the CA1 neurons more susceptible to excitotoxic cell death [55], [56].

Although the precise mechanisms leading to neurodegeneration in AD remain unclear, many studies indicate that Aβ could play a role in cell death by inducing mitochondrial oxidative stress and other processes [35], [57]. In this study, we have shown that there is a correlation between cell death in the CA1 region of the hippocampus and total Aβ expression, suggesting that Aβ may be contributing directly or indirectly to cell death in this region. Importantly, while neurodegeneration occurred in an age-dependent manner, and correlated strongly with the expression of total Aβ, cell loss was observed at least 12 weeks before the onset of plaques. Our data indicates that Aβ is present as early as 6 weeks of age and that this is most likely to be monomeric Aβ. Oligomeric Aβ formation appears at 24 weeks of age, and is significantly present by 36 weeks, forming along axons of neurons. Most importantly, plaque formation did not occur significantly until 36 weeks of age, indicating that plaque load is not the major driver of cell loss in this model of AD. While our study does not address current questions regarding the role of Aβ_40_ and Aβ_42_ [58], [59], it does suggest that plaque load need not be the major contributor to neurodegeneration, which begins at 12 weeks of age.

Inflammation is implicated in the etiology of AD. Many studies indicate that the release of pro-inflammatory cytokines from microglia and astrocytes can cause direct cell death of neurons both *in vivo* and *in vitro* [60], [61].We have shown, through quantitative analysis, that the numbers of CD68-positive microglia was significantly increased early in the hAPP-J20 mouse model. Our data also shows that accumulation of microglia correlates with cell death in the CA1 region of the hippocampus. Microglial accumulation around plaques has been extensively described in both AD patients and transgenic mouse models of AD and this is associated with elevation in cytokine levels. Our quantitative stereological analysis revealed significant increases in activated (CD68-positive) microglia prior to Aβ plaque deposition. In addition, the change in CD68-positive microglia significantly correlates with the extent of CA1 neuronal cell loss. Our data raises the possibility that imaging microgliosis might offer an approach to monitor AD progression in humans. Interestingly, astrogliosis also begins in these mice, starting at 12 weeks of age, though plateaus later in the disease progress. Other AD models have shown age-dependent increases in astrogliosis, though it is not clear why this phenomenon occurs in the hAPP-J20 model. Nonetheless, our results are consistent with recent patient data, which showed no correlation between microgliosis and astrogliosis with plaque load [62].

The correlation between Aβ and microglia, and microglia and neuronal cell death in the CA1 region of the hippocampus, supports the theory that monomeric and oligomeric Aβ causes the activation of microglia, which in turn is able to release pro-inflammatory cytokines, stimulating toxic signaling pathways and contributing to cell death [13], [63], [64]. Many pro-inflammatory cytokines have been shown to directly contribute to neurodegeneration and, in parallel, molecules secreted from neurons can promote further inflammatory processes [65]. This order of events is consistent with the literature on the pro-inflammatory cytokines that these cells secrete controlling the promoter activity of the APP gene [66], thus upregulating production of APP generation in many tissues, including brain [67], [68], [69], [70], [71], [72], [73]. Thus, as these processes are occurring prior to plaque onset in the hAPP-J20 mouse model, it is possible that neurodegeneration could be occurring due to a cycle of inflammation and neurodegeneration that further promotes inflammation. In this model activation of inflammatory cells, either by Aβor by other mechanisms is a key initiating event in AD that leads to a cycle of neurodegeneration and further inflammation [13], [74], [75], [76].

Behavioral impairments are a major constituent of AD and are readily described in mouse models of AD. Previous studies have characterized the hAPP-J20 mouse model using the Morris Water Maze (MWM) [39], [44], [45], [77], however interpretation has been confounded by variable results in the cued version of the MWM [28], [78]. Therefore, in this study, spatial memory and learning was investigated using the RAM. The RAM provides an advantage over the MWM as the hAPP-J20 mouse model has a high tendency to float and trend for thigmotactic swimming [43], [45]. In addition, the MWM can result in physical fatigue and hypothermia, which does not occur in the RAM. Furthermore, the RAM takes advantage of the animals’ natural food exploratory behavior. Our analysis of learning and memory by RAM revealed that the hAPP-J20 mice display decreased learning and memory in a hippocampal-dependent spatial memory task. Specifically, we have revealed that spatial reference memory deficits occur in the RAM at 16 and 24 weeks of age in the hAPP-J20 mouse model. Moreover, we also found impairments in long-term memory occurred 14 days following the RAM training. Therefore, memory impairments occurred during the period that cell loss and neuroinflammation occurs, but well before the onset of plaques. These learning deficits seen in our study could not be due to increased motor activity, since hyperactivity would correspond to a decrease in the percentage of arms correct from the first session. As the percentage of correct arms was the same for both hAPP-J20 and WT at 16 and 24-weeks of age, this indicates that there is no correlation between hyperactivity and movement within the RAM. Importantly, vision is not affected in the hAPP-J20 model [79].

We also tested hAPP-J20 mice in a context fear-conditioning paradigm to assess short- and long-term hippocampal-dependent contextual memory. We found no deficits in contextual fear conditioning at 28 weeks and 36 weeks of age. In addition, no long-term contextual fear memory and learning deficits were detected in the fear-conditioning paradigm. The results are consistent with a recent study, which indicated spatial deficits but no deficits in fear conditioning in the hAPP-J20 model [43]. It is possible that compensatory mechanisms and/or alterations to functionality of the fear circuit may account for the lack of deficit, even in the absence of full hippocampal function [80], [81].

There has been significant debate about the best approach for tracking AD progression via PET [6]. Since our quantitative stereological approach shows that CD68 positive cell numbers correlate closely with loss of neuronal cell numbers and with Aβ expression, it is conceivable that a label of activated microglia may potentially aid as a marker to track progression of AD. Therefore our work raises a question as to whether PET imaging of a marker of activated microglia, while not diagnostic, might offer a useful way to track disease progression. Activated microglia can be detected *in vivo* using PET scan imaging, with the selective radioligand known as ^11^C-PK11195, which is known to correlate with levels of CD68-positive microglia [82]. Indeed, PET scanning in a small cohort of patients with mild cognitive impairments (MCI) has revealed the presence of activated microglia [83]. ^11^C-PK11195 labelling is significantly increased in AD patients [84], [85], [86] and animal models [82]. In addition ^11^C-PK11195-labelled activated microglia has been shown to correlate with AD patient Mini-Mental State examination scores [85]. Since MCI has been shown to be a precursor for early AD there is therefore a considerable need to further investigate microglial markers, such as CD68, for PET scanning in a larger sample size of very early AD patients. CD68-positive microglia, combined with other markers, may be ultimately utilized to track AD progression.

The consistent conclusion of our study, taken together with other studies [20], [34], [87], [88], is that behavioral decline, neuronal cell death and inflammatory cell activation precede plaque deposition, providing a strong indication that neurodegenerative processes are occurring independent of Aβ protein. Fundamentally this means that AD progressive decline may occur well before plaque deposition in patients. At present, the Aβ protein is often regarded as a central component to brain degradation in AD. As such, imaging studies and therapeutic targets [29], [89], [90] are largely based around decreasing Aβ deposition in the brain; and new techniques such as MRI and PET scanning for Aβ can only detect fibrillar forms, and are mostly directed at imaging plaques [29], [30]. In this study, we show that other hallmarks of AD, such as neuronal loss, neuroinflammation and behavioral deficits are vastly progressed before plaque onset in a mouse model of AD. Our study shows a correlation between activated microglia, Aβand neuronal cell loss, but not Aβ deposition in plaques, highlighting the potential importance that microglia may play in the early development of degeneration and cognitive decline in AD. Therefore the imaging of microglia using PET could be a useful indicator for the progression and early detection of AD.
